# Iranian Journal of Basic Medical Sciences: Pursuing the COPE membership

**DOI:** 10.22038/IJBMS.2023.21385

**Published:** 2023-01

**Authors:** Leila Arabi, Ali Roohbakhsh, Bizhan Malaekeh-Nikouei, BiBi Sedigheh Fazly Bazzaz

**Affiliations:** 1 Nanotechnology Research Center, Pharmaceutical Technology Institute, Mashhad University of Medical Sciences, Mashhad, Iran (Assistant Editor); 2 Pharmaceutical Research Center, Pharmaceutical Technology Institute, Mashhad University of Medical Sciences, Mashhad, Iran (Assistant Editor); 3 Biotechnology Research Center, Pharmaceutical Technology Institute, Mashhad University of Medical Sciences, Mashhad, Iran (Editor–in–Chief)

 According to Elsevier, Committee on Publication Ethics (COPE) is “a forum for editors of peer-reviewed journals to discuss issues related to the integrity of the scientific record”. In general, COPE helps journal editors handle ethical issues in publication. Historically, some medical journal editors founded COPE in 1997 to monitor the issues regarding misconduct in publication. The main goal was to make ethical practices “a normal part of the publishing culture”. More information about COPE is available at the following link: 

https://publicationethics.org/about/our-organisation

To reach the highest standards in publication ethics, journals and publishers need specific core practices recommended and published by COPE. In this regard, journals and publishers can find several cases and scenarios with advice, complete guidance with practical resources, and education units to help and support them comply with their policies and decision-making.

These “Core Practices” which apply to editors and their journals, publishers, and institutions were established and published in 2017. The different areas regarding the “Core Practices” are listed in Table 1. The full description of each area (including the case database, guidelines, flowcharts, etc.) is available in the following website: 

https://publicationethics.org/core-practices

In May 2021, the Iranian Journal of Basic Medical Sciences (IJBMS) applied for COPE membership. Our application is currently in the COPE membership subcommittee for final review. Being a member of COPE confirms and ensures that IJBMS aims to follow the designated standards of publication ethics. In this regard, we have attempted in recent years to increase the transparency of the “Guide for authors” section in our website, diversify and expand our editorial board members, and follow the policies of COPE when dealing with related evolving ethical issues. Our editorial team tried to deliver timely responses to the challenging issues in accordance with the COPE guidelines. For instance, we have dealt with some special cases during 2018, which were addressed in a successful manner (1).

In the end, we would like to take this opportunity to recognize our colleagues’ continued hard work and dedication to IJBMS and our reviewers’ assistance. Their contribution is crucial for improving our journal’s quality, credibility, and integrity. It is important to thank all authors for choosing to publish with us. Their contribution to the advancement of basic medical research is highly valued.

We wish our readers and colleagues a healthy and safe NEW YEAR. Our aim is to continuously improve our offering and services to our audience community in the coming year.

**Table 1:** The main “Core Practices” for publication developed in 2017 by COPE

Allegations of misconduct
Authorship and contributorship
Complaints and appeals
Conflicts of interest
Data and reproducibility
Ethical oversight
Intellectual property
Journal management
Peer review processes
Post-publication discussions
